# Explainable Machine Learning Model for Predicting Postoperative Survival in Patients With Locally Advanced Gastric Cancer

**DOI:** 10.1002/cam4.71408

**Published:** 2025-11-21

**Authors:** Zhijie Gong, Liping Zhou, Yinghao He, Yanjie Deng, Jun Zhou, Weiwei Wang, Qiangbang Yang, Jian Pan, Yingze Li, Xiaolu Yuan, Minghui Ma

**Affiliations:** ^1^ The First School of Clinical Medicine Southern Medical University Guangzhou China; ^2^ Second Department of Gastrointestinal Surgery Maoming People's Hospital Maoming China; ^3^ Thyroid and Vascular Surgery Department Maoming People's Hospital Maoming China; ^4^ The First Clinical College of Medicine Guangdong Medical University Zhanjiang China; ^5^ Department of Pathology Maoming People's Hospital Maoming China

**Keywords:** explainable machine learning, locally advanced gastric cancer, random survival forest, survival prediction

## Abstract

**Purpose:**

This study aims to develop and validate an explainable machine learning model for predicting postoperative survival in patients with locally advanced gastric cancer (LAGC), optimizing predictive accuracy while ensuring clinical applicability to facilitate personalized prognostication for patients.

**Methods:**

The study utilized data from 8616 LAGC patients who underwent gastrectomy (2004–2015) in the Surveillance, Epidemiology, and End Results (SEER) database for model development and validation, with external validation performed using 235 postoperative LAGC cases (2016–2022) from Maoming People's Hospital (Maoming, China). Five predictive models—Cox proportional hazards model (CoxPH), random survival forest (RSF), extreme gradient boosting (XGBoost), gradient boosting machine (GBM), and DeepSurv—were developed using the training set. Model performance was evaluated using the concordance index (C‐index), area under the receiver operating characteristic curve (AUROC), and Brier score. Additionally, 1‐, 3‐, and 5‐year receiver operating characteristic curves (ROC), calibration curves, and decision curve analysis (DCA) were employed for further assessment. The optimal model was interpreted using explainability tools such as SurvSHAP and SurvLIME. Finally, an interactive prediction tool was created to provide personalized survival evaluation for LAGC patients.

**Results:**

RSF exhibited the highest predictive performance, with a C‐index of 0.732 (95% CI: 0.720–0.745) in the validation set and 0.723 (95% CI: 0.696–0.755) in the external validation set. The 1‐, 3‐, and 5‐year AUROCs were 0.771, 0.803, and 0.809 in the validation set, and 0.802, 0.711, and 0.721 in the external validation set. Explainability analysis identified lymph node ratio (LNR), AJCC stage, and age as the most influential prognostic factors. An interactive prediction tool was developed to provide individualized prognosis visualization.

**Conclusion:**

This study developed an RSF‐based model to predict postoperative survival in LAGC patients, emphasizing the prognostic significance of LNR, AJCC stage, and age. The interactive prediction tool enhances clinical utility, facilitating personalized treatment decision‐making for physicians.

## 1 Introduction

Gastric cancer (GC) ranks as the fifth most frequently diagnosed malignancy worldwide and the third leading cause of cancer‐related deaths [1]. Due to the absence of specific early symptoms, the majority of patients are diagnosed at locally advanced or advanced stages [2]. For those diagnosed with locally advanced gastric cancer (LAGC), surgery combined with adjuvant therapy remains the primary treatment approach [3, 4]. However, the overall postoperative survival rate remains unsatisfactory, with a 5‐year survival rate below 30% [5, 6, 7]. The interplay of multiple factors, including tumor staging, molecular subtypes, and individual patient heterogeneity, significantly impacts prognosis, posing a considerable challenge for accurate prognostic prediction [8]. The American Joint Committee on Cancer (AJCC) staging system is the predominant prognostic evaluation instrument for LAGC in clinical practice. While it provides guidance for treatment decisions, it does not offer sufficient prognostic information [9]. Therefore, developing an accurate predictive model for postoperative survival in LAGC patients is essential to optimize clinical decision‐making and enhance patient outcomes.

Machine learning (ML), a core branch of artificial intelligence, empowers algorithms to autonomously identify patterns within data and develop predictive models [10]. Traditional linear statistical models have been extensively used in predicting postoperative survival in LAGC patients, demonstrating a certain degree of utility [11, 12, 13]. However, in real‐world clinical practice, considering only linear relationships among clinical variables is insufficient. Compared to traditional statistical methods, ML demonstrates substantial advantages in handling high‐dimensional, nonlinear, and complex interactions in data, enabling the integration of multimodal clinical data to enhance predictive accuracy [14]. However, the “black‐box” nature of machine learning models presents difficulties in interpreting the dynamic contributions of key prognostic factors to individual survival risk, potentially undermining clinicians' trust in the predictive outcomes [15]. In recent years, various explainable artificial intelligence (XAI) algorithms have been introduced [16], striking a balance between predictive accuracy and interpretability. For patients with LAGC, explainable machine learning models for predicting postoperative survival remain scarce.

This study integrates data from the Surveillance, Epidemiology, and End Results (SEER) database and Maoming People's Hospital (Maoming, China) to develop and validate an explainable ML‐based model for predicting postoperative survival in LAGC patients. The model is designed to optimize predictive accuracy while ensuring clinical applicability, offering personalized prognostic insights for both clinicians and patients.

## 2 Materials and Methods

### 2.1 Data Collection

**FIGURE 1:**
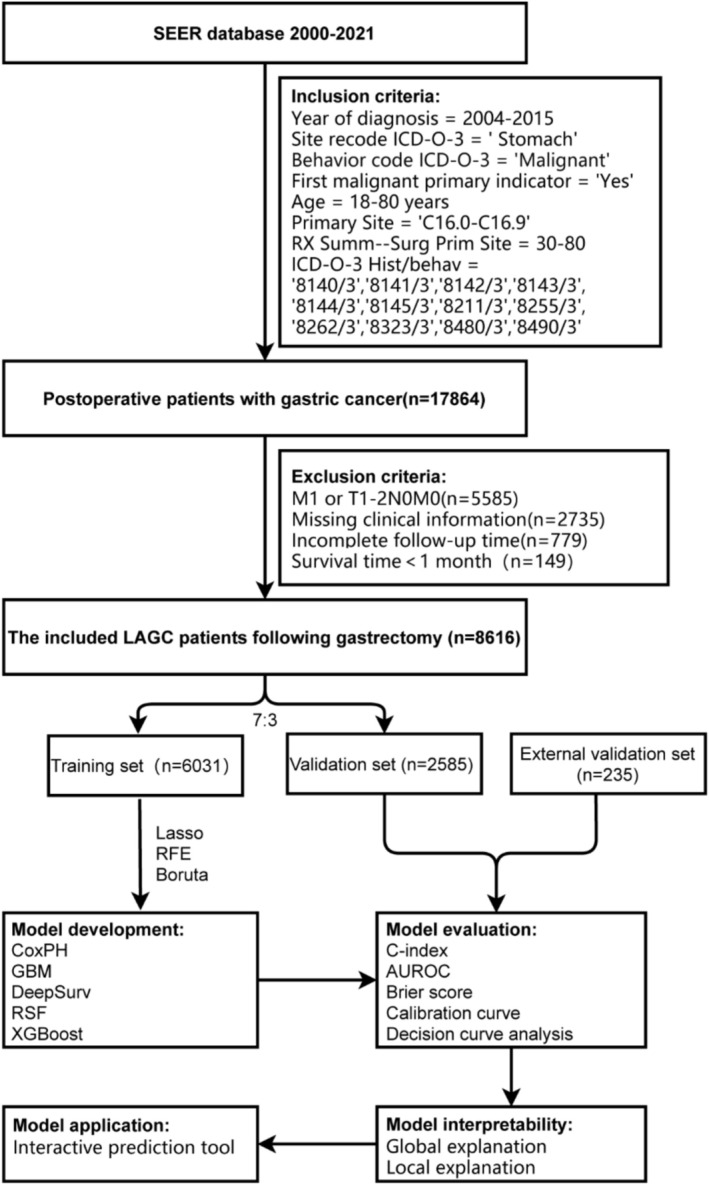
Flowchart of the research.

This study was a retrospective analysis, with the overall workflow illustrated in Figure 1. Data were extracted from the SEER Research Data (17 Registries, Nov 2023 submission, 2000–2021) using SEER*Stat software (v8.4.4), selecting cases diagnosed between 2004 and 2015 according to the specified inclusion criteria: (i) age between 18 and 80 years; (ii) primary gastric malignancies (anatomical location: C16.0–C16.9; ICD‐O‐3 histological codes: 8140/3–8490/3); and (iii) patients who underwent gastrectomy (RX Summ‐Surg Prim Site codes 30–80). Exclusion criteria were: (i) distant metastatic (M1) or early‐stage gastric cancer (T1‐2N0M0); (ii) cases with missing key clinical variables or incomplete follow‐up data; and (iii) overall survival (OS) < 1 month. Additionally, an external validation set of 235 LAGC patients treated at Maoming People's Hospital (Maoming, China) between 2016 and 2022 was included, complying with the identical inclusion and exclusion criteria. The study was approved by the Ethics Committee of Maoming People's Hospital (approval number: PJ2025MI‐K038‐01). Given that the study utilized only de‐identified historical data, the ethics committee waived the requirement for informed consent.

### 2.2 Variables

Patient survival data (overall survival and the corresponding endpoint event status) and clinical characteristics were systematically collected, including demographic variables (sex, age, race, marital status), tumor characteristics (location, size, grade, and histological type), staging details (TNM stage standardized according to the 8th edition of the AJCC classification), lymph node metastatic burden (number of positive lymph nodes and total examined lymph nodes), and treatment modalities (extent of surgery and receipt of chemotherapy or radiotherapy). Univariate Cox regression analyses were conducted for all variables, and those with *p* < 0.05 were incorporated into multivariate Cox regression analysis to ascertain independent prognostic factors.

A multi‐stage feature selection strategy was employed, where clinical variables were screened using Least Absolute Shrinkage and Selection Operator (LASSO), Recursive Feature Elimination (RFE), and the Boruta algorithm. The final feature set comprised consensus variables identified through the intersection of all three methods. LASSO is an embedded feature selection method that applies L1 regularization, shrinking the coefficients of non‐informative variables to zero, effectively handling multicollinearity and reducing overfitting [17]. RFE is a feature selection technique that employs a backward elimination strategy to iteratively refine the feature subset [18]. In this study, we propose a GBM‐RFE variant, which utilizes gradient boosting machine (GBM) to determine feature importances and iteratively remove the least important features, making it well‐suited to capturing complex or nonlinear relationships. Both LASSO and RFE were implemented with five‐fold cross‐validation to enhance selection stability. The Boruta algorithm, a wrapper‐based feature selection method, introduces “shadow features” as benchmark references. A random forest model then computes *Z*‐scores for real and synthetic noise features, applying statistical hypothesis testing to determine feature importance [19].

### 2.3 Model Development

Patients from the SEER database were randomly assigned to a training set and a validation set in a 7:3 ratio. Five representative predictive models were developed using the training set: Cox proportional hazards model (CoxPH), random survival forest (RSF), gradient boosting machine (GBM), extreme gradient boosting (XGBoost), and DeepSurv.

CoxPH is a classical semi‐parametric survival analysis method that establishes a log‐linear relationship between covariates and the hazard function using partial likelihood estimation, offering an interpretable hazard ratio framework. RSF, a non‐parametric ensemble learning approach, constructs multiple survival trees and employs splitting criteria to maximize survival differences across nodes, effectively capturing nonlinear effects and interactions [20]. GBM iteratively optimizes an additive model by minimizing the negative log‐likelihood loss function, enabling adaptive learning for heterogeneous survival data [21]. XGBoost extends GBM by incorporating L1/L2 regularization, second‐order gradient approximation, and parallel computation. Through feature binning and a sparsity‐aware algorithm, it enhances computational efficiency, rendering it especially appropriate for high‐dimensional survival prediction tasks [22]. DeepSurv, a deep learning‐based survival model, utilizes neural networks to capture high‐order feature interactions through nonlinear transformations in hidden layers, enabling the modeling of complex survival risk patterns [23]. Hyperparameters for all machine learning models were optimized using grid search with five‐fold cross‐validation, evaluating performance by the concordance index (C‐index).

### 2.4 Model Evaluation

Multiple metrics were utilized to evaluate model performance. C‐index and integrated area under the receiver operating characteristic curve (Integrated AUROC) assessed global discriminatory ability, while 1‐year, 3‐year, and 5‐year ROC plots were plotted to evaluate time‐specific discrimination. Typically, higher C‐index and AUC values indicate superior discriminative ability. Integrated Brier Score (IBS) and calibration curves were utilized to evaluate predictive accuracy. An IBS < 0.25 was considered indicative of an acceptable overall prediction error, while calibration curves were used to visualize local calibration bias across risk strata, with closer alignment to the diagonal reflecting lower bias. Decision curve analysis (DCA) was performed to quantify the net benefit of each model at different probability thresholds, providing insights into clinical utility. The integrated AUROC was calculated as the inverse‐probability‐of‐censoring‐weighted (IPCW) mean of the time‐dependent AUROC values at 1, 3, and 5 years, and the IBS was calculated similarly.

Risk categories (high‐, medium‐, and low‐risk) were established using tertile cutoffs derived from risk scores of the optimal model. Prognostic distinctions among these groups were evaluated using Kaplan–Meier survival analysis with log‐rank tests.

### 2.5 Model Explainability and Application

A multi‐level explainability framework was implemented to elucidate the predictions of the optimal model. At the global level, we generated time‐dependent feature‐importance plots, partial dependence survival profiles, and SurvSHAP(t) (a Shapley Additive Explanations variant for survival) plots. These plots were used to assess the contributions of individual features to model predictions and to illustrate how these contributions evolve over survival time. At the local level, case‐specific explanations were provided through SurvSHAP(t) plots and Survival Local Interpretable Model‐agnostic Explanations (SurvLIME) plots, enabling visualization of individualized prediction mechanisms.

Finally, an interactive application was created based on the optimal model to facilitate personalized postoperative survival prediction for LAGC patients, providing a user‐friendly platform for clinicians and patients.

### 2.6 Statistical Analysis

All statistical analyses and data visualizations were performed using R (version 4.3.3). Categorical variables were compared using the chi‐square test, while continuous variables were analyzed using the Mann–Whitney U test. Survival prediction models were developed using the “mlr3proba” framework, with DeepSurv implemented via the reticulate interface to call the Pycox module. Model explainability was conducted using the “survex” package, and the interactive prognostic prediction platform was built using the Shiny framework. A *p* value < 0.05 was deemed statistically significant.

## 3 Results

### 3.1 The Characteristics of Patients

A total of 8616 LAGC patients from the SEER database and 235 patients from Maoming People's Hospital who underwent gastrectomy were included in the study. Table 1 summarizes the baseline characteristics of patients, highlighting notable differences between the SEER and Chinese cohorts. Regarding demographic data, racial composition differed entirely between the two cohorts, and the percentage of married patients was markedly greater in the China cohort compared to the SEER dataset (95.7% vs. 64.2%, *p* < 0.001). Regarding tumor characteristics, the proportion of T4 stage cases was significantly higher in the China cohort than in the SEER dataset (68.5% vs. 33.7%, *p* < 0.001). Additionally, while 55.3% of patients in the SEER dataset were diagnosed at stage III, the proportion was even higher in the China cohort (65.5%), indicating that patients in the China cohort were diagnosed at a more advanced tumor stage. In terms of treatment modalities, the China cohort had a lower percentage of patients receiving chemotherapy or radiotherapy. Notably, only a very small proportion of patients in the China cohort received radiotherapy. Furthermore, discrepancies were noted in N staging, tumor location, histological type, surgical scope, and the quantity of lymph nodes examined between the two datasets.

**TABLE 1:** The characteristics of LAGC patients in SEER and China cohort.

Variables	SEER cohort			China cohort	*P*
	Training set	Validation set	Total	External validation set	
	(*n* = 6031)	(*n* = 2585)	(*n* = 8616)	(*n* = 235)	
Age (years)	62.72 ± 11.48	62.56 ± 11.74	62.67 ± 11.56	62.19 ± 11.43	*0.473*
Sex					*0.420*
Male	3956 (65.6%)	1671 (64.6%)	5627 (65.3%)	147 (62.6%)	
Female	2075 (34.4%)	914 (35.4%)	2989 (34.7%)	88 (37.4%)	
Race					< 0.001***
White	3984 (66.1%)	1719 (66.5%)	5703 (66.2%)	0 (0%)	
Black	824 (13.7%)	331 (12.8%)	1155 (13.4%)	0 (0%)	
Others^a^	1223 (20.3%)	535 (20.7%)	1758 (20.4%)	235 (100%)	
Marital					< 0.001***
Married	3890 (64.5%)	1643 (63.6%)	5533 (64.2%)	225 (95.7%)	
Unmarried	822 (13.6%)	372 (14.4%)	1194 (13.9%)	2 (0.9%)	
Others^b^	1319 (21.9%)	570 (22.1%)	1889 (21.9%)	8 (3.4%)	
T stage					< 0.001***
T1	350 (5.8%)	169 (6.5%)	519 (6%)	3 (1.3%)	
T2	470 (7.8%)	225 (8.7%)	695 (8.1%)	5 (2.1%)	
T3	3204 (53.1%)	1296 (50.1%)	4500 (52.2%)	66 (28.1%)	
T4a	1524 (25.3%)	678 (26.2%)	2202 (25.6%)	154 (65.5%)	
T4b	483 (8%)	217 (8.4%)	700 (8.1%)	7 (3%)	
N stage					0.027*
N0	1385 (23%)	550 (21.3%)	1935 (22.5%)	52 (22.1%)	
N1	1475 (24.5%)	671 (26%)	2146 (24.9%)	59 (25.1%)	
N2	1426 (23.6%)	626 (24.2%)	2052 (23.8%)	74 (31.5%)	
N3a	1252 (20.8%)	517 (20%)	1769 (20.5%)	39 (16.6%)	
N3b	493 (8.2%)	221 (8.5%)	714 (8.3%)	11 (4.7%)	
AJCC Stage					< 0.001***
IB	210 (3.5%)	101 (3.9%)	311 (3.6%)	0 (0%)	
IIA	1373 (22.8%)	550 (21.3%)	1923 (22.3%)	28 (11.9%)	
IIB	1117 (18.5%)	498 (19.3%)	1615 (18.7%)	53 (22.6%)	
IIIA	1462 (24.2%)	668 (25.8%)	2130 (24.7%)	50 (21.3%)	
IIIB	1276 (21.2%)	499 (19.3%)	1775 (20.6%)	88 (37.4%)	
IIIC	593 (9.8%)	269 (10.4%)	862 (10%)	16 (6.8%)	
Size (cm)	5.31 ± 2.96	5.3 ± 2.99	5.31 (2.97)	5.01 ± 2.39	*0.331*
Location					< 0.001***
Cardia/Fundus	1712 (28.4%)	744 (28.8%)	2816 (32.7%)	31 (13.2%)	
Body	535 (8.9%)	208 (8%)	743 (8.6%)	61 (26%)	
Antrum/Pylorus	1951 (32.3%)	865 (33.5%)	2456 (28.5%)	133 (56.6%)	
Curvature^c^	1004 (16.6%)	404 (15.6%)	1408 (16.3%)	6 (2.6%)	
Overlapping/NOS	829 (13.7%)	364 (14.1%)	1193 (13.8%)	4 (1.7%)	
Grade					*0.155*
Well	166 (2.8%)	53 (2.1%)	219 (2.5%)	3 (1.3%)	
Moderately	1476 (24.5%)	601 (23.2%)	2077 (24.1%)	69 (29.4%)	
Poorly	4249 (70.5%)	1883 (72.8%)	6132 (71.2%)	160 (68.1%)	
Undifferentiated	140 (2.3%)	48 (1.9%)	188 (2.2%)	3 (1.3%)	
Histology					< 0.001***
Adenocarcinoma	4293 (71.2%)	1794 (69.4%)	6087 (70.6%)	203 (86.4%)	
SRCC	1259 (20.9%)	614 (23.8%)	1873 (21.7%)	24 (10.2%)	
Others^d^	479 (7.9%)	177 (6.8%)	656 (7.6%)	8 (3.4%)	
Surgery					< 0.001***
Partial	4083 (67.7%)	1773 (68.6%)	5856 (68%)	173 (73.6%)	
Total	1433 (23.8%)	593 (22.9%)	2026 (23.5%)	62 (26.4%)	
Gastrectomy, NOS	515 (8.5%)	219 (8.5%)	734 (8.5%)	0 (0%)	
Radiation					< 0.001***
No/Unknown	2923 (48.5%)	1268 (49.1%)	4191 (48.6%)	229 (97.4%)	
Yes	3108 (51.5%)	1317 (50.9%)	4425 (51.4%)	6 (2.6%)	
Chemotherapy					< 0.001***
No/Unknown	1719 (28.5%)	761 (29.4%)	2480 (28.8%)	105 (44.7%)	
Yes	4312 (71.5%)	1824 (70.6%)	6136 (71.2%)	130 (55.3%)	
RNE	19.17 ± 12.54	19.57 ± 13.25	19.29 ± 12.76	15.33 ± 8.37	< 0.001***
LNR	0.3 ± 0.3	0.3 ± 0.3	0.3 ± 0.3	0.28 ± 0.27	*0.523*

*Note:* Values are shown as a number (%) or mean ± SD. SD denotes standard deviation. *p* value from comparisons between the total SEER cohort and the China cohort. (*p < 0.05, **p < 0.01, ***p < 0.001).

^a^ Asian/Pacific Islander and Indian/Alaska Native.

^b^ Widowed/Separated/Divorced.

^c^ Lesser or Greater Curvature.

^d^ Carcinoma, diffuse type; Linitis plastica. SRCC, signet ring cell carcinoma. RNE, number of regional nodes examined. LNR, lymph node ratio.

### 3.2 Variable Selection

**FIGURE 2:**
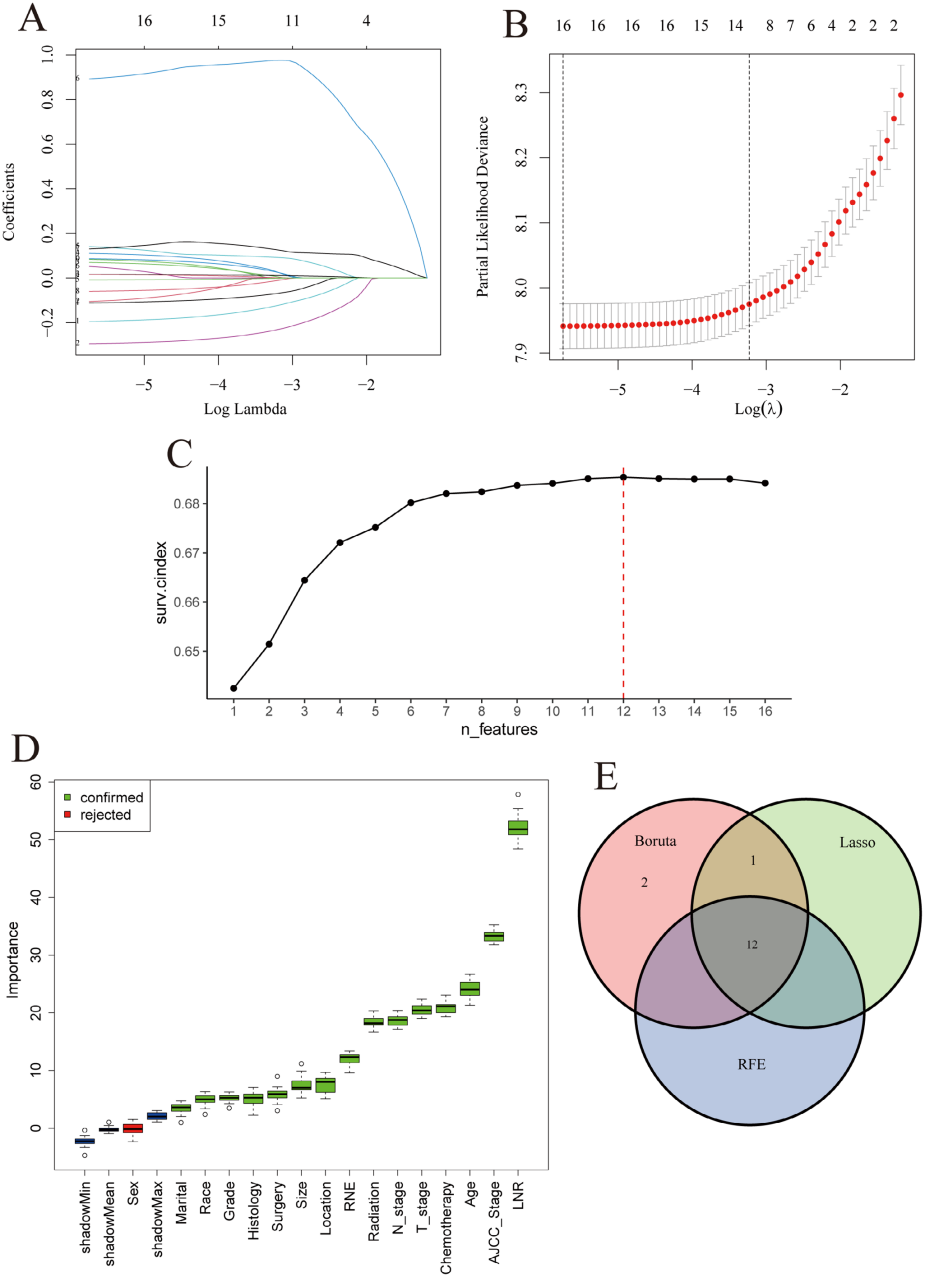
Feature Selection Results. (A) LASSO coefficient profiles. (B) Cross‐validation for LASSO showing the optimal lambda. (C) Recursive Feature Elimination (RFE) curve with the optimal feature count. (D) Feature importance ranking from the Boruta algorithm. (E) Venn diagram of selected features from LASSO, RFE, and Boruta.

Table 2 presents the results of the Cox regression analysis. Multivariate Cox regression revealed that all variables, except for sex and AJCC stage, were independent prognostic factors. Subsequently, three feature selection methods—LASSO, RFE, and the Boruta algorithm—were applied to clinical variables (Figure 2). LASSO regression identified 13 variables, while Boruta and RFE selected 15 and 12 variables, respectively. The intersection of these methods yielded 12 key prognostic features: age, race, T stage, AJCC stage, tumor size, tumor location, histological grade, RNE (number of regional nodes examined), LNR (lymph node ratio; defined as positive lymph nodes/total lymph nodes examined), extent of surgery, chemotherapy, and radiotherapy. These features were incorporated into the model development process.

**TABLE 2:** Univariate analysis and multivariate analysis.

Variable	Univariate analysis		Multivariate analysis	
	HR (95% CI)	*p*	HR (95% CI)	*p*
Age	1.02 (1.01–1.02)	< 0.001***	1.02 (1.01–1.02)	< 0.001***
Sex				
Male	Reference			
Female	0.95 (0.89–1.01)	*0.088*	—	—
Race				
White	Reference		Reference	
Black	0.96 (0.88–1.05)	*0.399*	0.99 (0.90–1.08)	*0.757*
Others^a^	0.78 (0.72–0.84)	< 0.001***	0.80 (0.74–0.87)	< 0.001***
Marital				
Married	Reference		Reference	
Unmarried	1.05 (0.96–1.15)	*0.281*	1.07 (0.97–1.17)	*0.163*
Others^b^	1.23 (1.15–1.32)	< 0.001***	1.12 (1.04–1.20)	0.003**
T stage				
T1	Reference		Reference	
T2	1.31 (1.10–1.57)	0.003**	1.09 (0.85–1.41)	*0.503*
T3	1.68 (1.45–1.94)	< 0.001***	1.39 (1.03–1.88)	0.031*
T4a	2.41 (2.08–2.80)	< 0.001***	1.66 (1.19–2.32)	0.003**
T4b	2.89 (2.44–3.42)	< 0.001***	2.03 (1.28–3.20)	0.003**
N stage				
N0	Reference		Reference	
N1	1.27 (1.16–1.39)	< 0.001***	1.26 (1.08–1.48)	0.004**
N2	1.67 (1.52–1.82)	< 0.001***	1.28 (1.02–1.59)	0.031*
N3a	2.35 (2.14–2.57)	< 0.001***	1.48 (1.01–2.17)	0.043*
N3b	3.47 (3.09–3.89)	< 0.001***	1.71 (0.97–3.03)	*0.066*
AJCC stage				
IB	Reference		Reference	
IIA	1.24 (1.02–1.52)	0.032*	1.25 (0.92–1.70)	*0.158*
IIB	1.75 (1.43–2.14)	< 0.001***	1.40 (0.99–1.97)	*0.059*
IIIA	2.36 (1.94–2.87)	< 0.001***	1.51 (0.99–2.29)	*0.056*
IIIB	3.11 (2.55–3.78)	< 0.001***	1.50 (0.87–2.60)	*0.145*
IIIC	4.97 (4.04–6.10)	< 0.001***	1.73 (0.83–3.59)	*0.143*
Size	1.06 (1.05–1.07)	< 0.001***	1.01 (1.00–1.02)	0.016*
Location				
Cardia/Fundus	Reference		Reference	
Body	0.84 (0.75–0.94)	0.002**	0.69 (0.62–0.78)	< 0.001***
Antrum/Pylorus	0.92 (0.85–0.99)	0.028*	0.76 (0.70–0.82)	< 0.001***
Curvature^c^	0.82 (0.75–0.90)	< 0.001***	0.70 (0.63–0.76)	< 0.001***
Overlapping/NOS	1.12 (1.02–1.23)	0.013*	0.78 (0.71–0.86)	< 0.001***
Grade				
Well	Reference		Reference	
Moderately	1.10 (0.91–1.33)	*0.347*	1.08 (0.89–1.31)	*0.410*
Poorly	1.42 (1.18–1.71)	< 0.001***	1.21 (1.00–1.46)	*0.051*
Undifferentiated	1.55 (1.19–2.01)	0.001***	1.43 (1.10–1.87)	0.008**
Histology				
Adenocarcinoma	Reference		Reference	
SRCC	1.23 (1.15–1.32)	< 0.001***	1.20 (1.11–1.29)	< 0.001***
Others^d^	1.18 (1.06–1.32)	0.003**	1.13 (1.01–1.26)	0.039*
Surgery				
Partial	Reference		Reference	
Total	1.21 (1.13–1.29)	< 0.001***	1.12 (1.04–1.20)	0.004**
Gastrectomy, NOS	1.25 (1.13–1.38)	< 0.001***	1.11 (1.00–1.24)	*0.057*
Radiation				
No/Unknown	Reference		Reference	
Yes	0.72 (0.68–0.77)	< 0.001***	0.83 (0.77–0.89)	< 0.001***
Chemotherapy				
No/Unknown	Reference		Reference	
Yes	0.66 (0.62–0.71)	< 0.001***	0.72 (0.66–0.77)	< 0.001***
RNE	0.99 (0.99–1.00)	< 0.001***	0.99 (0.99–0.99)	< 0.001***
LNR	4.54 (4.14–4.98)	< 0.001***	2.42 (2.03–2.90)	< 0.001***

*Note:* (*p < 0.05, **p < 0.01, ***p < 0.001).

^a^ Asian/Pacific Islander and Indian/Alaska Native.

^b^ Widowed/Separated/Divorced.

^c^ Lesser or Greater Curvature.

^d^ Carcinoma, diffuse type; Linitis plastica. SRCC, signet ring cell carcinoma. RNE, number of regional nodes examined. LNR, lymph node ratio.

### 3.3 Model Comparisons

Five prediction models were evaluated in the validation and external validation sets. The model performance metrics are summarized in Table 3. Among these models, RSF exhibited superior performance with C‐index values of 0.732 (95% CI: 0.720–0.745) and 0.723 (95% CI: 0.696–0.755), Integrated AUROC of 0.794 and 0.757, and Integrated Brier scores of 0.171 and 0.172 in the validation and external validation cohorts, respectively, demonstrating superior discrimination and calibration.

**TABLE 3:** Performance metrics of five models.

Model	Training set			Validation set			External validation set		
	Integrated AUROC	Integrated brier score	C‐index	Integrated AUROC	Integrated brier score	C‐index	Integrated AUROC	Integrated brier score	C‐index
CoxPH	0.763	0.178	0.691	0.742	0.183	0.680	0.736	0.180	0.686
RSF	0.829	0.150	0.749	0.794	0.171	0.732	0.757	0.172	0.723
XGBoost	0.794	0.168	0.719	0.764	0.178	0.703	0.749	0.175	0.707
DeepSurv	0.767	0.178	0.696	0.749	0.182	0.687	0.746	0.169	0.696
GBM	0.786	0.191	0.711	0.743	0.204	0.682	0.725	0.248	0.688

**FIGURE 3:**
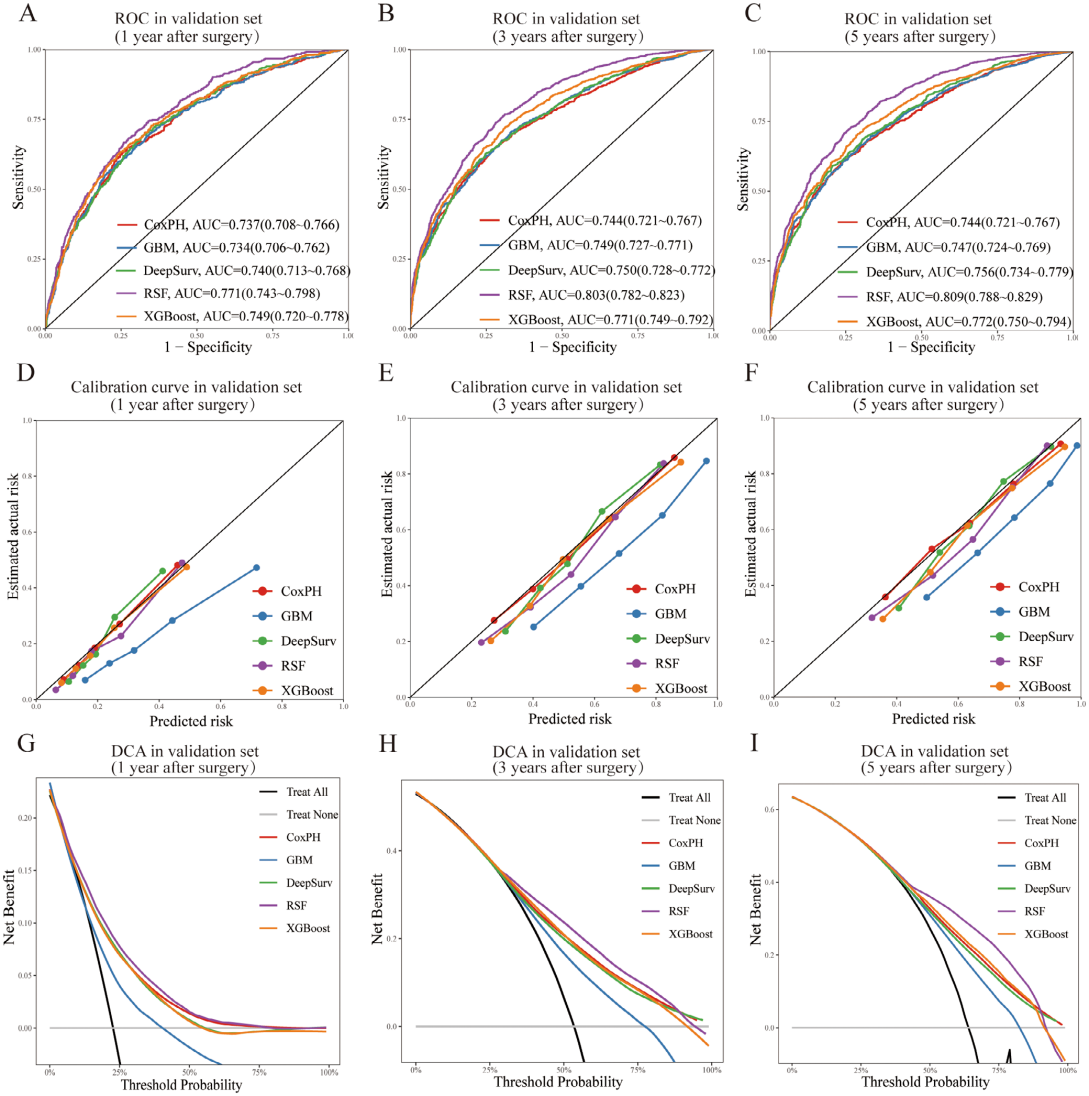
Model Performance Evaluation in the Validation Set. (A–C) ROC curves for 1‐, 3‐, and 5‐year survival predictions. (D–F) Calibration curves for 1‐, 3‐, and 5‐year survival, assessing prediction accuracy. (G–I) Decision curve analysis (DCA) for 1‐, 3‐, and 5‐year survival, evaluating the net clinical benefit of each model.

**FIGURE 4:**
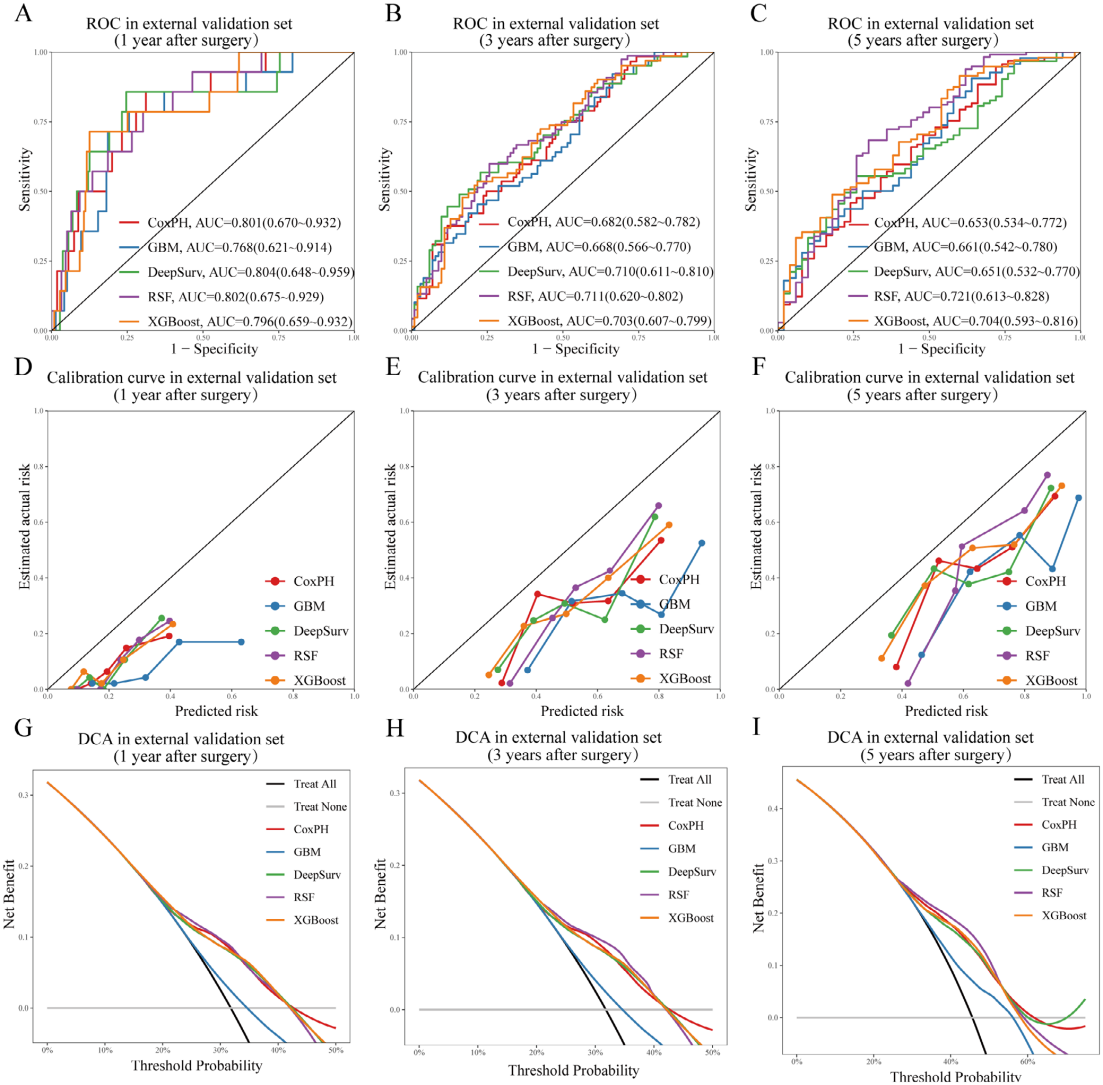
Model Performance Evaluation in the External Validation Set. (A–C) ROC curves for 1‐, 3‐, and 5‐year survival predictions. (D–F) Calibration curves for 1‐, 3‐, and 5‐year survival, assessing prediction accuracy. (G–I) Decision curve analysis (DCA) for 1‐, 3‐, and 5‐year survival, evaluating the net clinical benefit of each model.

ROC curves, calibration plots, and DCA were generated for all models (Supplementary Figure 1: training set; Figures 3 and 4: validation and external validation sets). RSF demonstrated superior discriminative performance, achieving higher AUROCs in both validation (1‐, 3‐, and 5‐year AUROCs: 0.771, 0.803, and 0.809) and external validation sets (1‐, 3‐, and 5‐year AUROCs: 0.802, 0.711, and 0.721). In the calibration plots, all models except GBM demonstrated good calibration in the validation set. Although calibration performance declined in the external validation set, RSF maintained relatively acceptable calibration. The DCA plot indicated that the RSF model provided a consistently higher net benefit than the other models across both validation cohorts over a broad range of threshold probabilities. Consequent to these findings, RSF was identified as the most suitable model for further investigation.

**FIGURE 5:**
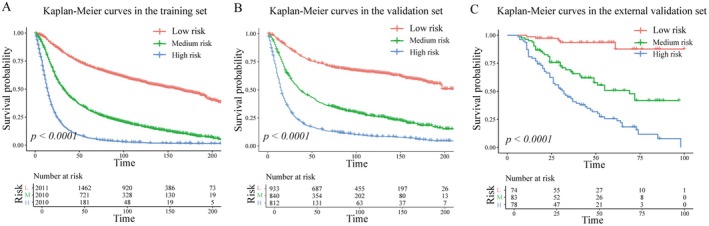
Kaplan–Meier curves for patients stratified into low‐, medium‐, and high‐risk groups based on RSF‐derived risk scores: (A) Training set; (B) Validation set; (C) External validation set.

A risk stratification system was constructed using RSF‐derived risk scores (Figure 5), which effectively classified patients into high‐, medium‐, and low‐risk categories. Significant differences in prognosis were observed across these risk strata in all three cohorts (*p* < 0.001), demonstrating the model's robust prognostic stratification capability.

### 3.4 Global Explanation

**FIGURE 6:**
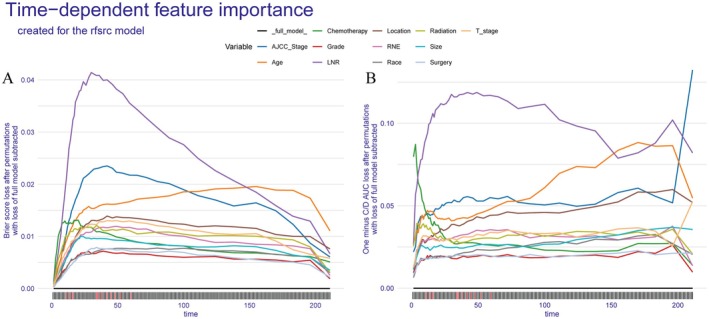
Time‐Dependent Feature Importance in the RSF Model. (A) Brier score loss after permuting each variable. (B) One‐minus C/D AUC loss after permuting each variable.

To enhance model interpretability, we began with a global interpretation of the model. As shown in Figure 6, we assessed time‐dependent variable importance for the RSF model using two distinct permutation‐based approaches—the Brier score loss and the C/D AUC loss. Greater changes in these loss metrics after permutation indicate higher importance of the respective clinical variables over time. LNR emerged as the most influential feature, particularly during the early survival period, with its importance gradually declining over time yet remaining significant in later survival stages. AJCC stage, T stage, and tumor location maintained high importance throughout the follow‐up period. Chemotherapy and radiotherapy played a crucial role in early survival predictions but exhibited diminishing effects in later periods. In contrast to LNR and AJCC stage, whose prognostic significance declined over time, the influence of age increased and ultimately exceeded both variables in later survival intervals.

**FIGURE 7:**
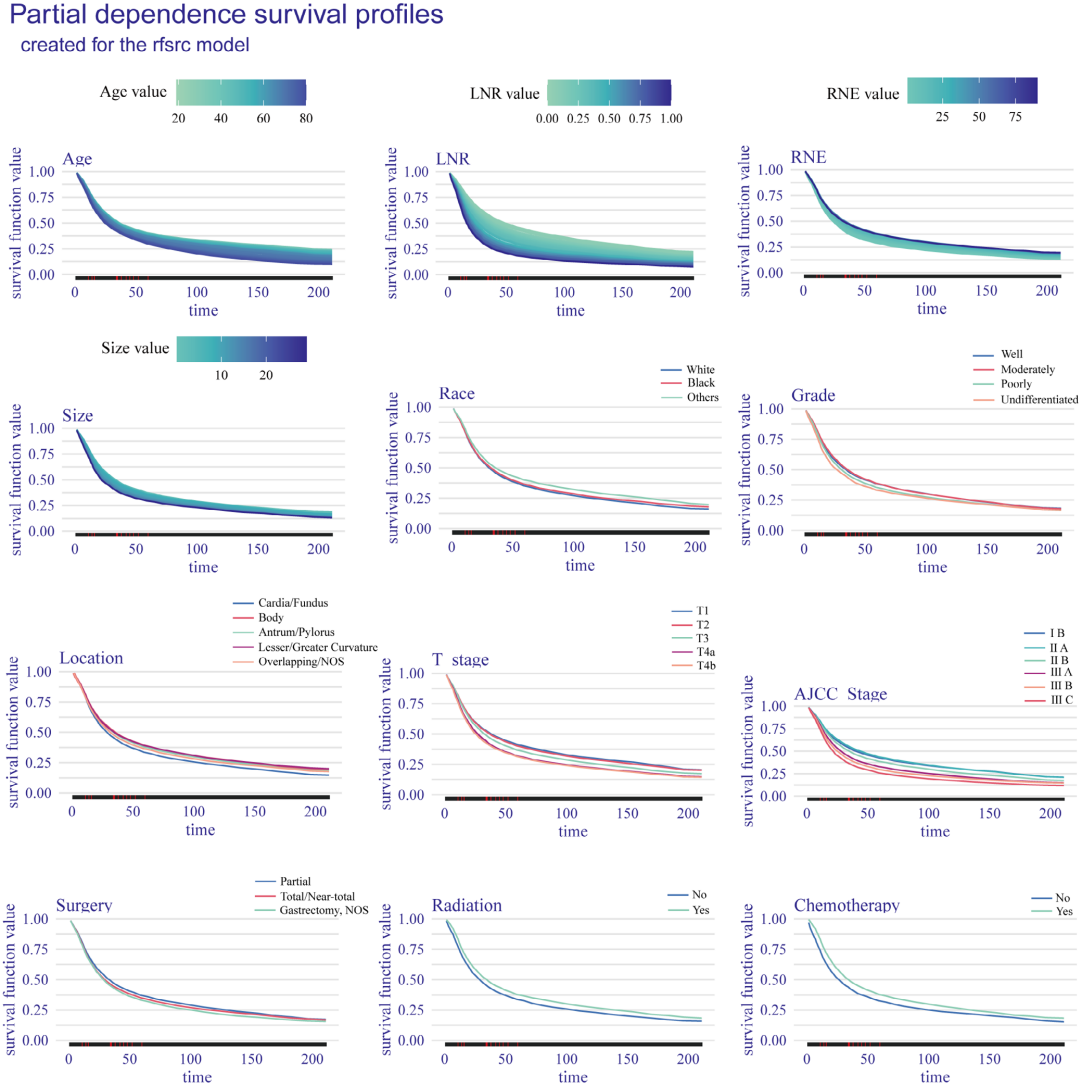
Partial Dependence Survival Profiles, illustrating the prognostic effects of each feature on survival over time.

Next, partial dependence survival curves were generated (Figure 7) to illustrate how variations in individual variables influenced overall survival while holding other variables constant. These curves confirmed that LNR, age, AJCC stage, and T stage were the most influential predictors of survival, with chemotherapy, radiotherapy, and tumor location also playing notable prognostic roles.

**FIGURE 8:**
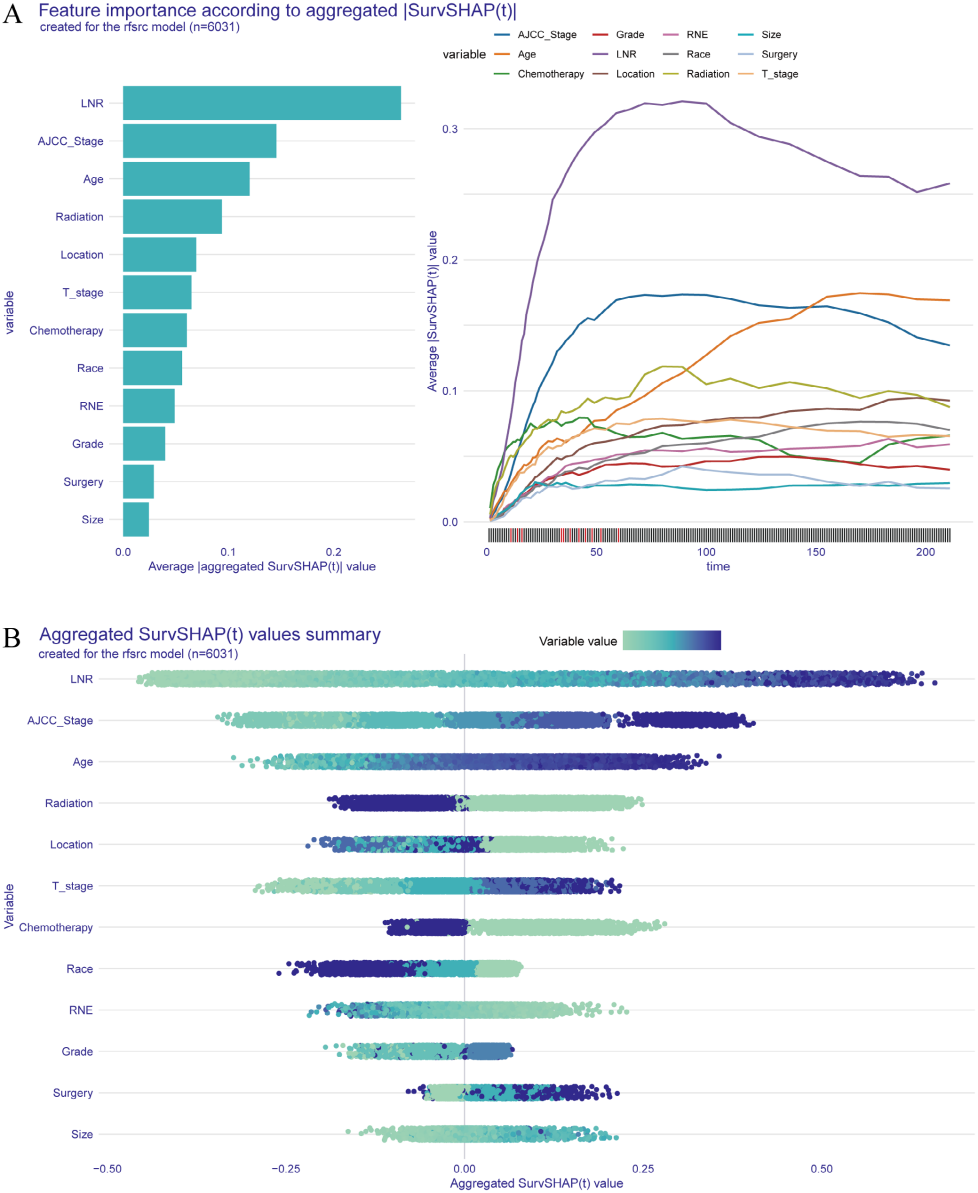
Global Explanation Using SurvSHAP(t). (A) Feature importance ranking based on aggregated SurvSHAP(t) values (left) and time‐dependent contribution trends of key variables (right). (B) Beeswarm plot illustrating the distribution of SurvSHAP(t) values across 6031 patients in the training set.

Finally, a SurvSHAP(t) summary plot was generated using RSF model predictions for 6031 patients in the training set (Figure 8). SurvSHAP(t), an explainable artificial intelligence (XAI) methodology for survival analysis, extends the classical SHAP framework by quantifying feature contributions across the survival time dimension [24]. Figure 8A (left) presents a bar plot ranking the aggregated absolute SurvSHAP(t) values (|SurvSHAP(t)|), indicating variable importance. LNR was the most influential predictor, followed by AJCC stage, age, and radiotherapy. Tumor location, T stage, and chemotherapy also played significant roles, while tumor size, histological grade, and extent of surgery had relatively lower importance. The right panel of Figure 8A displays time‐dependent trends of variable importance, showing that LNR was crucial for short‐term survival prediction, whereas age and tumor location became more important for long‐term survival, consistent with the time‐dependent feature importance plot in Figure 6.

The beeswarm plot in Figure 8B further visualizes the distribution of SurvSHAP(t) values across 6031 patients. Variables are arranged by importance, and the horizontal axis represents SurvSHAP(t) values, with larger values indicating greater impact on survival risk. The color intensity represents variations in the variables: for continuous variables, a transition from light to dark indicates an increase in value, while for categorical variables, different colors represent different categories. This visualization further supports the conclusion that LNR, AJCC stage, and age are key prognostic predictors.

### 3.5 Local Explanation

**FIGURE 9:**
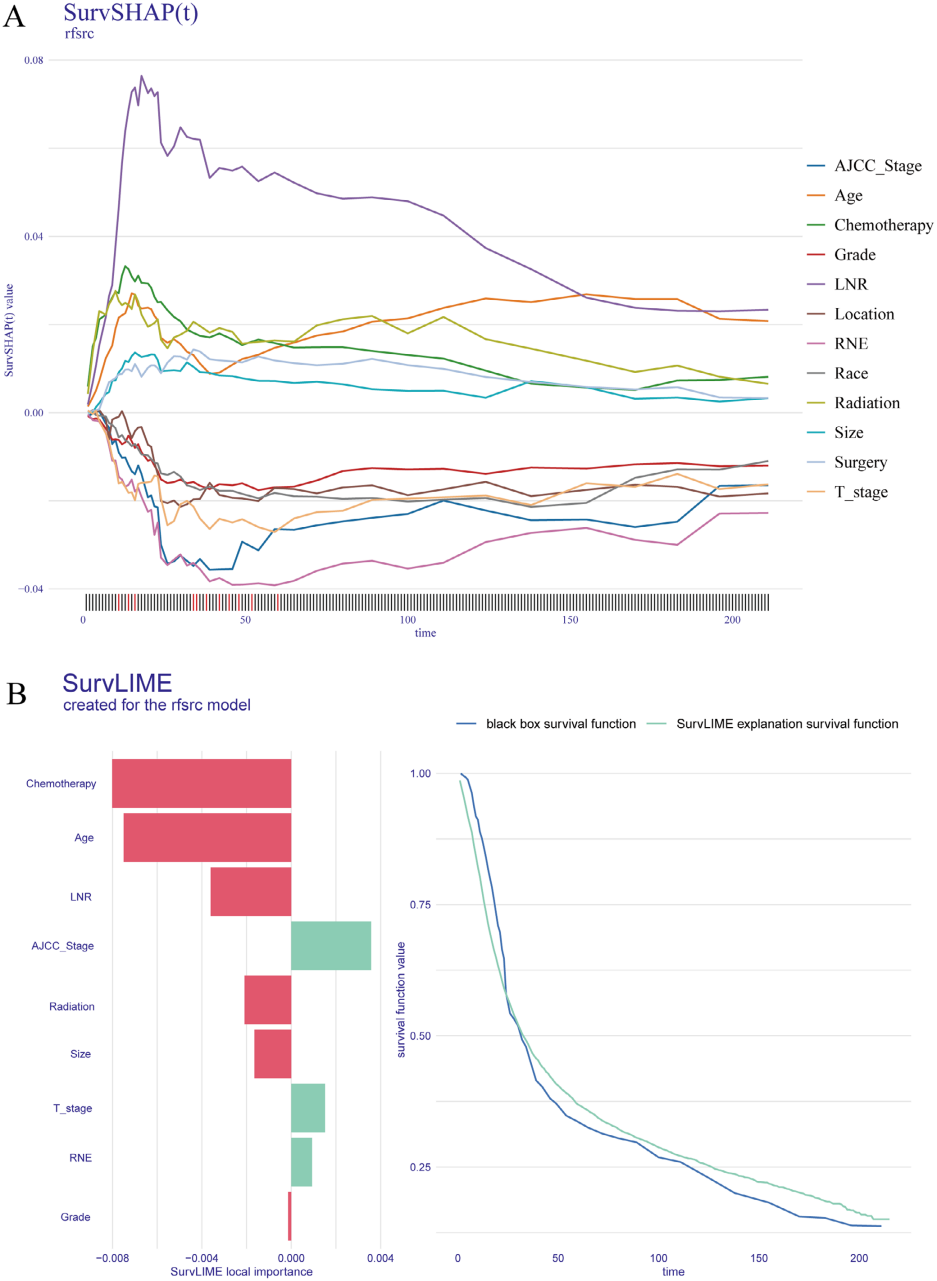
Local Explanation Using SurvSHAP(t) and SurvLIME. (A) Dynamic contributions of prognostic features over time for an individual patient based on SurvSHAP(t). (B) SurvLIME analysis demonstrating local feature importance (left) and comparison between “black‐box” model and its interpretable SurvLIME approximation (right).

SurvSHAP(t) also enables individualized explanations for specific patients. As shown in Figure 9A, we selected patient #5581, whose risk score was at the median of the training set, for evaluation. This patient was a 56‐year‐old White individual with a 4 cm tumor in the cardia, pathologically staged as pT4aN1M0 (Stage IIIA), who underwent partial gastrectomy accompanied by chemoradiotherapy. The LNR was 0.09 and the RNE was 11. The vertical axis represents SurvSHAP(t) values, reflecting the relative contribution of each variable to survival prediction. Positive values denote an elevated survival probability, while negative values suggest a reduced survival probability. For this patient, a relatively lower LNR, younger age, and receipt of chemoradiotherapy contributed to improved survival, whereas advanced T and AJCC staging, along with inadequate lymph node examination, were associated with a reduced survival probability.

SurvLIME, an adaptation of the LIME algorithm specifically for survival models, interprets individual predictions by locally perturbing input data and approximating the black‐box model behavior with a simpler linear model [25]. As shown in Figure 9B, the left panel displays variable contributions to this patient's survival prediction, with red bars denoting positive impacts and green bars denoting negative effects. Chemotherapy, age, LNR, and radiotherapy were the strongest positive predictors, while AJCC stage, T stage, and RNE were the strongest negative predictors, consistent with the SurvSHAP(t) results. The right panel of Figure 9B shows that the approximation model closely aligns with the black‐box model, enhancing the reliability of its interpretation.

### 3.6 The Individual Prognostic Prediction

**FIGURE 10:**
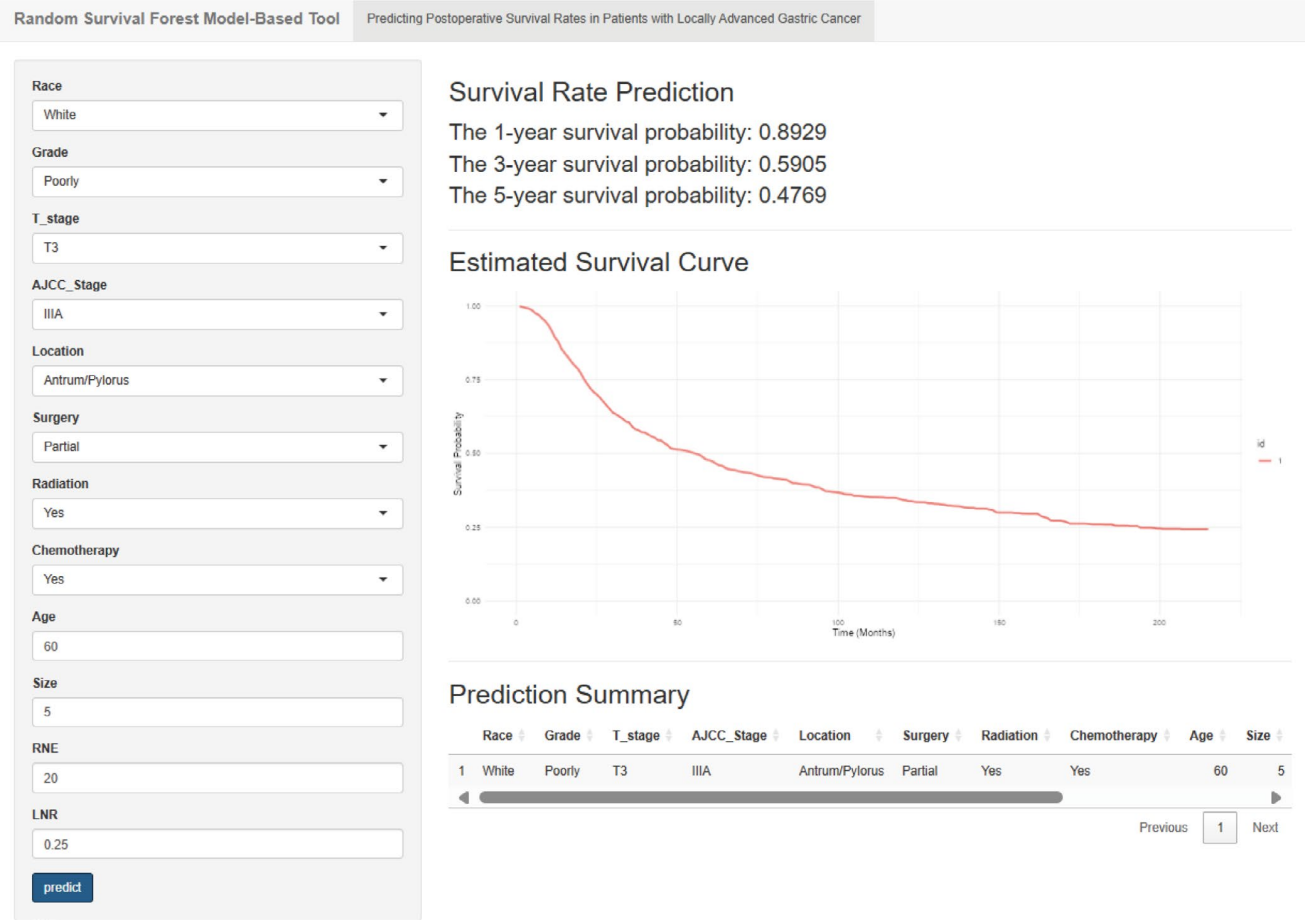
Interactive RSF‐Based Prognostic Prediction Tool. User interface for individualized survival prediction in LAGC patients.

An interactive prognostic prediction tool based on RSF was developed to provide personalized postoperative survival estimates for LAGC patients (Figure 10). The interface comprises two main sections: (1) a user input panel for entering clinical variables and (2) a results panel displaying survival probabilities and survival curves. The tool can be accessed on GitHub‐https://github.com/GZJ0526/LAGC. Download the required files from the website, launch the application, enter a patient's information, and click “Predict” to obtain the 1‐, 3‐, and 5‐year survival probabilities and the survival curve.

## 4 Discussion

LAGC is characterized by deep tumor infiltration, a high incidence of regional lymph node metastasis, and significant biological heterogeneity, resulting in inferior postoperative survival outcomes compared to early‐stage gastric cancer [8]. Accurate prognostic prediction in these patients is crucial for guiding clinical management. In fact, multiple prognostic models have been developed for predicting survival outcomes in patients with LAGC following surgery. For example, Sun et al. created a nomogram to predict survival in elderly patients with LAGC, achieving C‐indices of 0.687 and 0.713 in internal and external validation sets, respectively [11]. Yu et al. constructed a nomogram to predict survival in patients with locally advanced gastric signet ring cell carcinoma, reporting 1‐, 3‐, and 5‐year AUCs of 0.704, 0.759, and 0.767 in the validation set [13]. Although these nomogram‐based models incorporate multiple prognostic factors, they are constrained by the assumptions of linear models, limiting their ability to capture complex and dynamic interactions among clinical variables. Furthermore, traditional models rely on static predictions and cannot account for time‐dependent risks, reducing their utility in individualized and dynamic prognostic assessment.

With advancements in artificial intelligence, machine‐learning methods are increasingly applied to diagnostic classification and survival prognostication. Using data from 23,867 patients with colorectal cancer in the SEER program, Qiu et al. developed an XGBoost model to predict distant metastasis, achieving an AUC of 0.814 in an external validation cohort [26]. Zhao et al. leveraged the Survival Quilts framework to estimate post‐gastrectomy survival across multiple horizons (6 months to 10 years), with external‐validation C‐index ranging from 0.691 to 0.756 [27]. Beyond outcome prediction, machine learning also helps highlight key drivers of clinical outcomes. For example, in a random forest model predicting prolonged ICU length of stay after coronary artery bypass grafting, Jafarkhani et al. identified the duration of endotracheal intubation, body mass index, age, and operative time as the most influential predictors [28]. Likewise, a systematic review of prostate cancer studies highlighted age, prostate‐specific antigen (PSA), total PSA, free PSA, and PSA density as key risk indicators [29]. These studies can further help clinicians identify the clinical features most critical to disease progression, thereby guiding the optimization of treatment. However, machine learning models specifically designed for LAGC patients remain scarce. In this research, we utilized a multi‐method modeling technique that included (i) traditional statistical models (CoxPH), (ii) three major ML frameworks (RSF, XGBoost, and GBM), and (iii) deep learning architectures (DeepSurv). This comprehensive strategy systematically covers conventional statistical methodologies, state‐of‐the‐art ML techniques, and advanced neural network approaches. By comparing the performance of these models across internal and external validation datasets, RSF emerged as the optimal predictive model. RSF, an ensemble model composed of multiple survival trees, employs random feature selection and node‐splitting optimization strategies, enabling the capture of complex nonlinear effects without predefined risk function assumptions. RSF has been extensively utilized across multiple disease areas owing to its strong predictive efficacy [20, 30, 31].

Despite the strong predictive power of ML models, their “black‐box” nature presents a substantial obstacle to clinical use, as the lack of interpretability prevents clinicians from verifying the biological plausibility of model decisions. Even when models achieve excellent performance, their clinical utility remains limited if they do not provide interpretable decision rules or feature contribution rankings [32]. Efforts have been made to address this challenge through post hoc explainability frameworks such as LIME [33] and SHAP [34], which enhance interpretability while preserving model performance by utilizing local linear approximations or game‐theoretic feature attributions.

After identifying RSF as the best‐performing model, we conducted a multidimensional explainability analysis to examine the contributions and temporal dynamics of key prognostic variables. Among these variables, LNR emerged as the most important prognostic factor. LNR represents a comprehensive metric of both tumor lymphatic burden and the quality of lymphadenectomy. In our study, SHAP value distributions indicated that high LNR values were correlated with a markedly heightened risk of mortality. Prior research has likewise shown the predictive significance of LNR, showing that higher LNR values are strongly correlated with shorter survival in gastric cancer patients [35, 36, 37]. Additionally, our analysis revealed the time‐dependent nature of LNR's prognostic impact, with its influence being most pronounced in the early postoperative period but gradually diminishing over time. Mechanistically, a high LNR probably marks residual tumor burden and micrometastatic spread, which drive early relapse and cancer‐related death following gastrectomy [38, 39]. At the same time, high LNR patients who stay event free beyond the early postoperative period represent a selected subgroup with lower residual disease, in whom the later association between LNR and survival is attenuated. AJCC stage and T stage also exhibited significant prognostic value in the model, aligning with the widely accepted consensus that the AJCC staging system remains to be the widely used standard for evaluating prognosis in gastric cancer [40]. Notably, AJCC stage was significant in univariable analysis but was not retained in the multivariable Cox model. The most plausible explanation is collinearity with T and N stages, plus the limited flexibility of the standard Cox model to capture nonlinearity or interactions, leaving the AJCC stage with little incremental prognostic value. Age also emerged as a key prognostic factor, in line with prior research that has established advanced age as an independent prognostic factor in gastric cancer [12, 41]. It is noteworthy that age also exhibited a distinct time‐dependent effect. The increasing prognostic influence of age over time likely reflects growing competing risks in older patients. As cardiovascular disease, infections, accidental injuries, and other age‐related comorbidities accumulate during follow up, non‐cancer mortality contributes more to late deaths while tumor‐related hazards decline.

Regarding treatment, although the optimal timing and modality of chemoradiotherapy for gastric cancer remain controversial, its overall efficacy in improving patient survival has been well‐established by numerous studies [1, 42, 43, 44, 45]. Consistently, our model also identified chemoradiotherapy as a significant prognostic factor. Chemotherapy and radiotherapy exert their greatest influence in the early postoperative years by lowering the near‐term risk of recurrence. Patients who remain event free beyond this period form a selected subgroup with lower residual disease, so the treatment signal attenuates during later follow up. However, due to inherent limitations of the SEER database, key treatment‐related details—such as chemotherapy cycles and specific drug regimens—were unavailable, and the relatively small number of cases with complete radiotherapy data further constrained our ability to perform a more detailed treatment stratification analysis. Furthermore, categorizing untreated patients together with those whose treatment status was unknown might have introduced systematic bias into the evaluation of therapeutic efficacy, potentially resulting in either underestimation or overestimation of the actual impact of chemoradiotherapy on clinical outcomes. Additional research is required to refine the prognostic role of chemoradiotherapy.

The interactive prognostic prediction tool puts the model into practice. Using routine postoperative data, the RSF tool produces a patient‐specific survival curve and 1‐, 3‐, and 5‐year probabilities. Clinicians can use these data to plan treatment and follow‐up schedules. High‐risk patients receive closer monitoring and more frequent visits, which may improve survival.

This research possesses several limitations. First, As SEER does not include important features such as vascular invasion, perineural invasion, and molecular markers, the model lacks these predictors, which can lower discrimination, introduce miscalibration in specific subgroups, and weaken transportability across centers. Second, the external validation cohort was retrospective, single center, and relatively small, which limits the demonstration of generalizability. The model's robustness may be affected by unavoidable selection bias or residual overfitting. In the future, we plan to build a large multicenter prospective cohort with comprehensive molecular, pathologic, and treatment information to support model development and validation.

## 5 Conclusions

In conclusion, this study developed five ML‐based models using SEER and single‐center Chinese data to predict postoperative survival in LAGC patients, identifying RSF as the best‐performing model in both validation and external validation cohorts. Through global and local explainability analyses, we identified LNR, AJCC stage, and age as key prognostic drivers and elucidated their time‐dependent effects. The RSF‐based interactive prediction tool facilitates individualized prognosis visualization, offering an accurate and practical decision‐support system for dynamic clinical management, with the potential to optimize risk stratification strategies in gastric cancer.

## Author Contributions

**Zhijie Gong:** data curation, formal analysis, project administration, writing – original draft, writing – review and editing. **Liping Zhou:** data curation, formal analysis, project administration, writing – review and editing, writing – original draft. **Yinghao He:** data curation, methodology. **Yanjie Deng:** data curation. **Jun Zhou:** data curation. **Weiwei Wang:** data curation. **Qiangbang Yang:** data curation. **Jian Pan:** data curation. **Yingze Li:** data curation. **Xiaolu Yuan:** project administration, writing – review and editing. **Minghui Ma:** project administration, writing – review and editing.

## Ethics Statement

The study was approved by the Ethics Committee of Maoming People's Hospital (approval number: PJ2025MI‐K038‐01). Informed consent to participate was not applicable for this retrospective study.

## Conflicts of Interest

The authors declare no conflicts of interest.

## Supporting information


**Data S1:** Supporting Information.

## Data Availability

The data that support the findings of this study are available from the corresponding author upon reasonable request.
