# Supplementary material for: Explainable Machine Learning Model for Predicting Postoperative Survival in Patients With Locally Advanced Gastric Cancer
Source: Cancer Med. 2025 Nov 21;14(22):e71408. doi: 10.1002/cam4.71408 (PMC12638127; doi:10.1002/cam4.71408)
Supplement: Supplementary file 1 — Data S1: Supporting Information. [file CAM4-14-e71408-s001.docx]

**
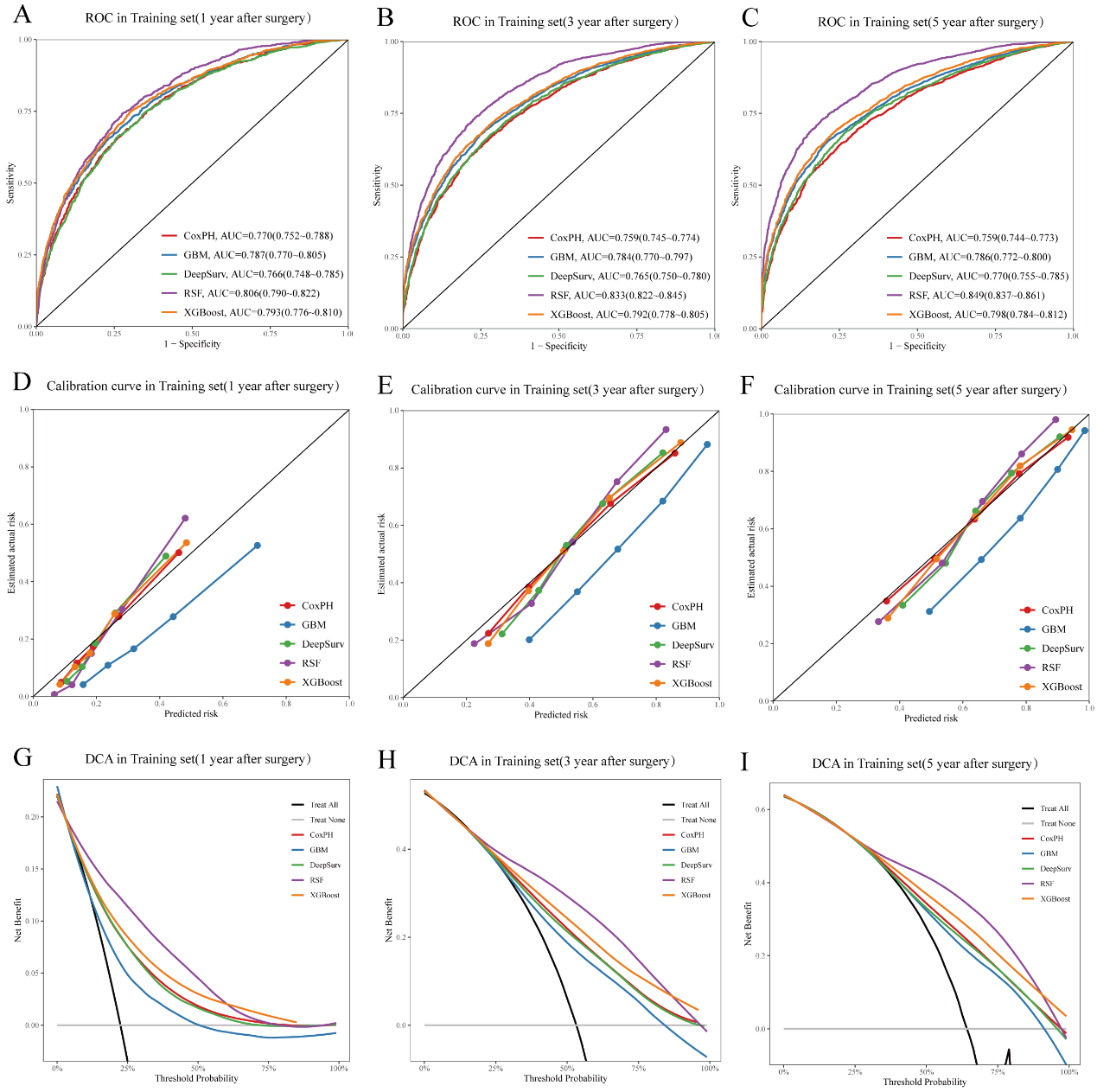
**

**Supplementary Figure 1**：Model Performance Evaluation in the training set. (A-C) ROC curves for 1-, 3-, and 5-year survival predictions. (D-F) Calibration curves for 1-, 3-, and 5-year survival, assessing prediction accuracy. (G-I) Decision curve analysis (DCA) for 1-, 3-, and 5-year survival, evaluating the net clinical benefit of each model.
